# Sweet Cherry Fruit: Ideal Osmometers?

**DOI:** 10.3389/fpls.2019.00164

**Published:** 2019-03-05

**Authors:** Andreas Winkler, Eckhard Grimm, Moritz Knoche

**Affiliations:** Fruit Science Section, Institute of Horticultural Production Systems, Leibniz-University Hannover, Hannover, Germany

**Keywords:** prunus avium, water uptake, cuticle, water potential, osmotic potential, reflection coefficient

## Abstract

Osmotic water uptake through the skin is an important factor in rain cracking of sweet cherries. The objective was to establish whether a sweet cherry behaves like an ideal osmometer, where: (1) water uptake rates are negatively related to fruit osmotic potential, (2) a change in osmotic potential of the incubation solution results in a proportional change in water uptake rate, (3) the osmotic potential of the incubation solution yielding zero water uptake is numerically equal to the fruit water potential (in the absence of significant fruit turgor), and (4) the fruits' cuticular membrane is permeable only to water. The fruits' average osmotic potential and the rate of water uptake were related only weakly. Surprisingly, incubating a fruit in (a) the expressed juice from fruit of the same batch or (b) an isotonic artificial juice composed of the five major osmolytes of expressed juice or (c) an isotonic glucose solution—all resulted in significant water uptake. Decreasing the osmotic potential of the incubation solution decreased the rate of water uptake, while decreasing it still further resulted in water loss to the incubation solution. Throughout fruit development, the “apparent” fruit water potential was always more negative than the fruits' measured average osmotic potential. Plasmolysis of epidermal cells indicates the skin's osmotic potential was less negative than that of the flesh. When excised flesh discs were incubated in a concentration series of glucose solutions, the apparent water potential of the discs matched the osmotic potential of the expressed juice. Significant penetration of ^14^C-glucose and ^14^C-fructose occurred through excised fruit skins. These results indicate a sweet cherry is not an ideal osmometer. This is due in part to the cuticular membrane having a reflection coefficient for glucose and fructose less than unity. As a consequence, glucose and fructose were taken up by the fruit from the incubation solution. Furthermore, the osmotic potential of the expressed fruit juice is not uniform. The osmotic potential of juice taken from the stylar scar region is more negative than that from the pedicel region and that from the flesh more negative than that from the skin.

## Introduction

Rain cracking severely limits sweet cherry production in nearly all regions where this high-value crop is grown, and especially when rainfall occurs during the harvest period (Christensen, 1996). Osmotic water uptake through the fruit surface into the flesh is considered to be the primary cause of rain cracking (Christensen, 1996; Winkler et al., 2016). There is no published evidence that the pit plays any significant role in either water uptake or in rain cracking.

In the now familiar “osmometer” model, the fruit is regarded as a small, thin-walled vessel containing a sugary solution of negative osmotic potential (i.e., the semi-fluid flesh) surrounded by a semipermeable membrane (i.e., the cuticular membrane (CM)). As with an osmometer, the rate of osmotic water uptake (*F*_*H*_2_*O*_; g s^−1^) by a cherry fruit equals the product of the flux density (*J*; g m^−2^ s^−1^) times the surface area (*A*; m^2^). Meanwhile, *J* may be expressed as the product of the osmotic water permeability (*P*_*f*_; m s^−1^) and the water potential difference (ΔΨ) between the fruit (Ψ^*fruit*^) and the outside solution (${\text{Ψ}}_{\Pi }^{solution}$). Based on the gas laws, this is multiplied by the molar volume of water (*V*_*w*_) divided by the product of the gas constant (*R*) and the absolute temperature (*T*) ($\frac{V_{w}}{R\times T}$) (House, 1974; Kramer and Boyer, 1995; modified), hence we can write:

(1) $$\begin{array}{r} F_{H_{2}O}=J\times A=P_{f}\Delta \text{Ψ}\times A\times \frac{V_{w}}{R\times T} \end{array}$$

Since for rain water the value of ${\text{Ψ}}_{\Pi }^{solution}$ is very close to zero, the value of ΔΨ during rain is determined solely by Ψ^*fruit*^. Meanwhile, the value of Ψ^*fruit*^ equals the sum of a number of water potential components: the principal ones being the internal pressure [i.e., the fruit Ψ_*P*_ (relative to atmospheric pressure)], the average osmotic potential of the fruit tissue (${\text{Ψ}}_{\Pi }^{fruit}$) and the matric potential (Ψ_*M*_). Meanwhile, Ψ_*M*_ is a putative matric component but this is unlikely to occur in a ripe sweet cherry fruit as it contains no gas phase and thus no gas:liquid interfaces where surface (matric) tension could create a local Ψ_*M*_ component. Recent studies have established that values of fruit and cell turgor (Ψ_*P*_) of mature cherry fruit are negligibly low relative to values of ${\text{Ψ}}_{\Pi }^{fruit}$ (Knoche et al., 2014; Schumann et al., 2014). Thus, for a mature sweet cherry fruit either outside in the rain or inside immersed in pure water, we can safely introduce the simplification that ΔΨ is numerically equal to ${\text{Ψ}}_{\Pi }^{fruit}$.

From the above, one would expect close and positive relationships between *F*_*H*_2_*O*_ and *A, P*_*f*_ and ΔΨ, when a fruit is incubated in deionized water in the laboratory. Furthermore, expressing *F*_*H*_2_*O*_/*A* as the *J* eliminates *A* as a source of variability and this should yield close relationships of *J* with ${\text{Ψ}}_{\Pi }^{fruit}\;$ and/or *P*_*f*_. Also, by decreasing ΔΨ by increasing the osmolyte concentration in the incubation solution one would anticipate a simple proportional decrease in *F*_*H*_2_*O*_ and/or *J*. Furthermore, because osmotic activity is a colligative property, the relationship obtained should be independent of the osmolyte used.

To the best of our knowledge, relationships between *F*_*H*_2_*O*_ or *J* and ΔΨ or *P*_*f*_ have not been investigated in sweet cherry—or any other fruit crop bearing fleshy and juicy fruit. The only results we are aware of are in sweet cherry and reveal highly variable relationships between *F*_*H*_2_*O*_ (expressed as a % weight increase) and the value of ${\text{Ψ}}_{\Pi }^{fruit}$ (Christensen, 1972).

The objective of our study was to establish whether sweet cherry behaves like an ideal osmometer. To classify as an ideal osmometer, the following criteria should be fulfilled: (1) *F*_*H*_2_*O*_ and *J* should be negatively related to ${\text{Ψ}}_{\Pi }^{fruit}$, (2) a change in ${\text{Ψ}}_{\Pi }^{solution}$ should result in a corresponding change in *F*_*H*_2_*O*_, (3) the value of ${\text{Ψ}}_{\Pi }^{solution}\;$yielding zero net uptake should exactly match that of ${\text{Ψ}}_{\Pi }^{fruit}$ (in the absence of significant turgor) and should be independent of the osmolyte used, and (4) the CM should be permeable only to water but not to any osmolytes present in the fruit or in the incubation solution.

## Materials and Methods

### Plant Material

Sweet cherry fruit of the cultivars Adriana, Burlat, Hedelfinger, Merchant, Sam, Samba, Schneiders Späte Knorpel, and Regina and the sour cherry cultivars Achat, Morellenfeuer, and Ungarische Traubige were picked from greenhouse-grown or field-grown trees grafted on “Gisela 5” rootstocks (*P. cerasus* L. × *P. canescens* Bois) at the Horticultural Research Station of the Leibniz University in Ruthe, Germany (lat. 52°14′N, long. 9°49′E). Fruit were selected for uniformity of size, color, and freedom from defects.

The pedicels were cut flush with the receptacle. Pedicel end and receptacle as well as the pedicel/fruit junction were sealed using a fast curing silicone rubber (Dow Corning SE 9186; Dow Corning Corp., Midland, MI, USA). The silicone was allowed to cure for about 1 h. Sealing the pedicel/fruit junction restricted water uptake to the fruit surface (Beyer et al., 2002). All experiments were carried out in a temperature controlled laboratory at 22°C.

### Determining Water Uptake, the Apparent **Ψ*^fruit^*** and the Calculated **Ψ*_P_***

Water uptake and water loss were determined gravimetrically as changes in fruit mass. Fruit were weighed, incubated individually in various solutions, removed usually after 45 and 90 min, carefully dried using tissue paper, re-weighed and re-incubated. The value of *F*_*H*_2_*O*_ was calculated as the slope of a linear regression line fitted through a plot of cumulative water uptake vs. time. Fruits that cracked in the course of an experiment were excluded from the analyses.

The apparent Ψ^*fruit*^ was obtained by determining *F*_*H*_2_*O*_ from osmolyte solutions at concentrations ranging from hypertonic to hypotonic. We refer to the Ψ^*fruit*^ as the apparent Ψ^*fruit*^ because it is quantified indirectly by determining water uptake and loss. Briefly, a linear regression was fitted through a plot of *F*_*H*_2_*O*_ vs. the ${\text{Ψ}}_{\Pi }^{solution}$. Because these relationships were not linear, the regression line was limited to those data lying close to the x-axis intercept (i.e., close to the point of zero net change in mass). The intercept was calculated from the regression equation and corresponds to the ${\text{Ψ}}_{\Pi }^{solution}$ that resulted in constant fruit mass. Under these conditions, there is no net movement of water indicating that the apparent Ψ^*fruit*^ equals the ${\text{Ψ}}_{\Pi }^{solution}$ (Weichert and Knoche, 2006).

A fruit Ψ_*P*_ is classically calculated as the difference between Ψ^*fruit*^ and ${\text{Ψ}}_{\Pi }^{fruit}$ where the ${\text{Ψ}}_{\Pi }^{fruit}$ is typically determined on expressed juice by water vapor pressure osmometry (VAPRO® 5520 and 5600; Wescor, Logan, UT, USA) We refer to this Ψ_*P*_ as the calculated Ψ_*P*_.

### Determining the Osmotic Potential of the Skin

The osmotic potential of the skin (${\text{Ψ}}_{\Pi }^{skin}$) was determined using plasmolysis and the procedure described in detail by Grimm and Knoche (2015). Briefly, skin sections were prepared using a razor blade, the sections were momentarily rinsed in isotonic sucrose solution to remove any juice from the cut surface and blotted with tissue paper. The sections were immediately transferred to a glass microscope slide, covered with a cover slip and incubated in a sucrose solution (${\text{Ψ}}_{\Pi }^{solution}$) for 30 min by feeding the sucrose solution to the section on the microscope slide. The sucrose solutions used ranged in concentration from −5.0 to 0 MPa. Afterwards, the microscope slides were viewed at × 40 (BX-60; Olympus Europa, Hamburg, Germany) in transmitted light. Calibrated images were taken (DP73; Olympus). The numbers of plasmolyzed cells and the total numbers of cells were counted and the percentage of plasmolyzed cells was calculated. The values of ${\text{Ψ}}_{\Pi }^{fruit}$ and ${\text{Ψ}}_{\Pi }^{solution}$ were determined by water vapor pressure osmometry (VAPRO® 5520 and 5600; Wescor). The ${\text{Ψ}}_{\Pi }^{skin}$ was calculated using a sigmoidal regression model fitted through a plot of the percentage of plasmolyzed cells vs. the ${\text{Ψ}}_{\Pi }^{solution}$. The ${\text{Ψ}}_{\Pi }^{solution}$ at 50% plasmolysis represents the ${\text{Ψ}}_{\Pi }^{skin}$.

### Experiments

Relationships between ${\text{Ψ}}_{\Pi }^{fruit}$ and *F*_*H*_2_*O*_ from deionized water were investigated using “Adriana,” “Burlat,” “Hedelfinger,” “Merchant,” “Sam,” “Samba,” “Schneiders Späte Knorpel,” and “Regina” fruit. Fruit juice was extracted using a garlic press and the value of ${\text{Ψ}}_{\Pi }^{fruit}$ was determined (VAPRO® 5520 and 5600; Wescor). The number of individual fruits was 172. There were no consistent differences among cultivars, which allowed the data for all cultivars to be pooled.

The effect of varying ${\text{Ψ}}_{\Pi }^{solution}$ of artificial juice or of polyethylene glycol 6000 (PEG 6000; Merck Eurolab, Darmstadt, Germany) on *F*_*H*_2_*O*_ and on the apparent Ψ^*fruit*^ was quantified in “Adriana.” The artificial juice was made of the five major osmolytes present in real juice. The osmolytes in the artificial juice and their relative contributions to total osmolarity were: glucose (41.2%), fructose (37.5%), sorbitol (7.3%), malic acid (6.7%), and potassium as potassium malate (5.4%). These five osmolytes accounted for 98% of the osmotically active components of sweet cherry juice (Herrmann, 2001; Winkler et al., 2015). The artificial juice and the PEG 6000 solutions were prepared at values of ${\text{Ψ}}_{\Pi }^{solution}$ ranging from −5.1 to 0 MPa. The lowest (most negative) ${\text{Ψ}}_{\Pi }^{solution}$ were hypertonic relative to the ${\text{Ψ}}_{\Pi }^{fruit}$ and so resulted in negative rates of water uptake (i.e., water losses). Sweet cherry juice extracted from the same batch of fruit served as a control. The number of replicates was 10.

The effect of different cultivars on the calculated fruit Ψ_*P*_ was studied in “Adriana,” “Regina,” “Sam,” and “Samba.” Fruit were incubated in PEG 6000 solutions ranging from −5.4 to 0 MPa. Values of ${\text{Ψ}}_{\Pi }^{fruit}$ were determined by vapor pressure osmometry (VAPRO® 5520 and 5600; Wescor). The number of replicates was 10.

The effect of incubating fruit in expressed fruit juice on water uptake was studied in “Achat,” “Adriana,” “Hedelfinger,” “Morellenfeuer,” “Regina,” “Samba,” “Schneiders Späte Knorpel,” and “Ungarische Traubige”. Juice was extracted from the same batch of fruit. Values of ${\text{Ψ}}_{\Pi }^{fruit}$ were determined by vapor pressure osmometry (VAPRO® 5520 and 5600; Wescor). Fruit incubated in deionized water served as controls. The number of replicates was 10.

The effect of heat treatment to inactivate any enzymes present in the juice was studied in “Adriana”. Rates of water uptake were determined by incubating fruit in juice heated for 5 min to 60°C, then cooled to laboratory temperature before use. Juice that had not been heat-treated served as control. Juice was prepared from the same batch of fruit as described above. As an additional control, an isotonic artificial juice was prepared. Values of ${\text{Ψ}}_{\Pi }^{solution}$ were determined (VAPRO® 5520 and 5600; Wescor). The number of replicates was 15.

The effect of the pH of the sweet cherry juice on the rate of water uptake was investigated in “Regina”. Fruit were incubated in juice extracted from the same batch of fruit or in isotonic artificial juice. Both juices were used at their “native” pH (4.2) or titrated to pH 7 using KOH. The number of replicates was 15.

The effect of the developmental stage on the apparent Ψ^*fruit*^ was investigated in “Regina” at 32, 52, 71, and 91 days after full bloom (DAFB). The values of *F*_*H*_2_*O*_ from glucose solutions ranging in ${\text{Ψ}}_{\Pi }^{solution}$ from −5.8 to 0 MPa were determined. The number of replicates was 10.

To establish whether the history of transpiration of the fruit alters the apparent Ψ^*fruit*^, the effect of the water vapor concentration deficit during a holding period before an experiment was studied. Mature “Schneiders Späte Knorpel” fruit were preconditioned by incubation above dry silica gel (~0% RH) or above water (~100% RH) at 22°C for 4 d. Subsequently, the apparent Ψ^*fruit*^ was determined in glucose solutions (${\text{Ψ}}_{\Pi }^{solution}$) ranging from −4.9 to 0 MPa. Fresh fruit without a holding period and fruit held at ~100% RH for 4 d were used as controls. The number of replicates was 10.

To identify whether the water vapor concentration deficit during a holding period induced a gradient in ${\text{Ψ}}_{\Pi }^{fruit}$ within the fruit, “Adriana” fruit were allowed to transpire for up to 154 h above dry silica gel (~0% RH) and the mass loss quantified. Fruit incubated above water (~100% RH) served as controls. The values of ${\text{Ψ}}_{\Pi }^{fruit}$ from the flesh were determined. Tissue cylinders ranging in depth from the skin to the pit were excised from the equatorial region using a biopsy punch of 8 mm diameter. The cylinders were then cut into four discs, each of about 1.5 mm thick. The discs represented the entire transect of the flesh from skin to pit. The discs were squeezed to liberate their juice, and this was measured immediately (VAPRO® 5520 and 5600; Wescor). In a second experiment, plasmolysis of epidermal cells was quantified by incubating skin sections in sucrose solutions (${\text{Ψ}}_{\Pi }^{solution}$) ranging from −5.0 to 0 MPa (Grimm and Knoche, 2015). This procedure yields a high-resolution estimate of the ${\text{Ψ}}_{\Pi }^{skin}$ only. We are not aware of any other method to obtain this value. The history of transpiration of the fruit was varied. In one experiment, fruit were preconditioned by excluding any transpiration by incubation for up to 48 days above water (~100% RH at 22°C, “Regina”). In another experiment, fruit was incubated for 4 d above dry silica gel (~0% RH, 22°C, “Schneiders Späte Knorpel”) to maximize transpiration. Freshly sampled fruit were used as control. The number of replicates was 10.

The osmotic potentials of different parts of the fruit were analyzed in “Schneiders Späte Knorpel”. Fruit were cut in half along the pedicel/stylar scar axis. One half was squeezed and the juice used to determine the average osmotic potential of the fruit using a water vapor pressure osmometer (VAPRO® 5600, Wescor). This value represents the average osmotic potential of the whole fruit. The other half was cut horizontally (normal to the pedicel/stylar scar axis) into four equal slices (see Figure 5). The juice was squeezed from each slice and its osmotic potential was measured. The number of replicates was 10.

To identify whether the skin has an effect on the apparent Ψ^*fruit*^, the latter was determined by incubating flesh discs excised from ‘Regina' sweet cherry in glucose solutions differing in osmolarity. Using a biopsy punch, a flesh cylinder of 8 mm diameter was excised from the equatorial region of a fruit's cheek and another cylinder from the stylar scar region of the same fruit. The cylinders were then cut to form a series of 2 mm thick discs using parallel-mounted razorblades. The discs were incubated in multiwell plates (well diameter 24 mm) containing glucose at ${\text{Ψ}}_{\Pi }^{solution}$ ranging from −6.1 to 0 MPa. Calibrated digital photographs were taken after 0, 15 and 30 min of incubation (Canon EOS 550D, lens EFS 60 mm, f/2.8 Macro USM; Canon, Tokyo, Japan). The areas of the discs were quantified using image analysis software (cellSens 1.7.1; Olympus). The *F*_*H*_2_*O*_ values were calculated from the change in disc area during the first incubation interval. It is assumed the tissue was isotropic and, hence, the disc would have undergone a change in thickness (not measured), proportional to the change in area (measured). Whole fruit from the same batch and incubated in the same solutions served as controls. The minimum number of replicates was nine for discs and seven for whole fruit.

Penetration of ^14^C-glucose and ^14^C-fructose through the sweet cherry fruit skin was determined using the infinite dose technique (Bukovac and Petracek, 1993; Weichert and Knoche, 2006). Epidermal skin segments (ES; comprising cuticle, epidermis, hypodermis and several parenchyma layers) with diameters of 10 mm were excised using a biopsy punch and cut to a thickness of about 1 mm using a razor blade. The ES were blotted on tissue paper and mounted in lucite^R^ holders (inside diameter 5 mm) using silicon grease (Korasilon Paste hochviskos; Kurt Obermeier, Bad Berleburg, Germany) and the holders affixed between two glass half-cells with silicone-grease (Korasilon Paste hochviskos; Kurt Obermeier) such that the cuticle side of the ES faced the donor cell. A stirring bar was added to each half-cell and the cells placed on multiple stirring units. Diffusion cells were leak-checked by applying a slight hydrostatic pressure across the ES. Only those ES that maintained the gradient in hydrostatic pressure overnight were used in the experiment. The diffusion experiment was started by adding 5 ml of deionized water as receiver and 5 ml of donor solution containing ^14^C-glucose (1.9 × 10^4^ Bq ml^−1^) or ^14^C-fructose (1.4 × 10^4^ Bq ml^−1^). Specific activities were 10.8 GBq mmol^−1^ and 7.4 GBq mmol^−1^ for glucose and fructose, respectively. Aliquots of 1 ml were removed from the receiver cell at 0, 0.25, 0.5, 2, 4, 6, 23, and 30 h, radioassayed by liquid scintillation spectrometry (scintillation cocktail Ultima Gold XR; PerkinElmer, Waltham, MA, USA; counter: LS 6500; Beckman Instruments, Fullerton, CA, USA) and replaced by deionized water. The steady state flow rates were calculated on an individual ES basis by fitting a linear regression line through a plot of cumulative penetration vs. time (*F*_*CHO*_, Bq h^−1^). From the *F*_*CHO*_, the cross-sectional area of the ES exposed in the diffusion cell *A* (m^2^) and the difference in concentration of radioactivity between donor and receiver solutions ΔC (Bq m^−3^), the permeance (*P*) of the fruit skin to glucose and fructose was calculated according to:

(2) $$\begin{array}{r} P=\frac{F_{CHO}}{A\;\times \;\Delta C} \end{array}$$

In infinite dose diffusion, the amount of radioactivity penetrating into the receiver solution is extremely low (<0.3% of the donor) and, hence, ΔC remains essentially constant during the typical duration of an experiment. Thus, ΔC equals the concentration of radioactivity in the donor. The number of replicates was eight.

The permeances so obtained were used to predict uptake of glucose and fructose into mature sweet cherry fruit when incubated in a solution equivalent to the glucose or fructose concentration of the typical sweet cherry juice. The following equation was used:

(3) $$\begin{array}{r} F_{CHO}=P\times A\times \left(C_{do}-\;C_{rec}\right) \end{array}$$

In the above equation *C*_*do*_ represented the glucose or fructose concentration in the incubation solution and *C*_*rec*_ the initial concentrations in the fruit compartment or the skin compartment. The volume of the donor solution was assumed to be large relative to the fruit volume and hence, *C*_*do*_ remained nearly constant. In contrast, the receiver volume resembling the fruit or skin compartment only was small and *C*_*rec*_ changed as penetration proceeded. Typical concentrations in the juice are 0.432 M equiv. to 77.8 g l^−1^ for glucose and 0.394 M equiv. to 70.9 g l^−1^ for fructose (Herrmann, 2001). Glucose and fructose together account for 78.7% of the total osmolarity of the juice of a mature sweet cherry fruit. In the first simulation run, the fruit was assumed to be incubated in its own juice (*C*_*do*_ = 77.8 g l^−1^ or 70.9 g l^−1^ for glucose or fructose) and the entire fruit volume of a perfectly spherical, 10 g sweet cherry (equiv. to a fruit surface area *A* = 22.4 cm^2^) was used as the receiver. The initial internal concentrations of glucose and fructose were assumed to be zero (*C*_*rec*_ = 0 g l^−1^ for glucose and fructose). In the second run, uptake was simulated to occur into the fruit skin only. Skin thickness was estimated at 100 μm based on micrographs of cross-sections published by Glenn and Poovaiah (1989) and measurements of cell sizes (Brüggenwirth and Knoche, 2016). Simulations were carried out for a skin compartment containing no glucose or fructose (*C*_*rec*_ = 0 g l^−1^ for glucose and fructose) and subsequently for a skin compartment containing glucose initially at 0.233 M (equiv. to *C*_*rec*_ = 41.9 g l^−1^) and fructose initially at 0.212 M (equiv. to *C*_*rec*_ = 38.2 g l^−1^). These concentrations correspond to an average osmotic potential of fruit skin (−1.4 ± 0.1 MPa) relative to that of the flesh (−2.6 ± 0.2 MPa) as determined by plasmolysis in an earlier study (Grimm and Knoche, 2015).

### Data Analysis

**Figure 1:**
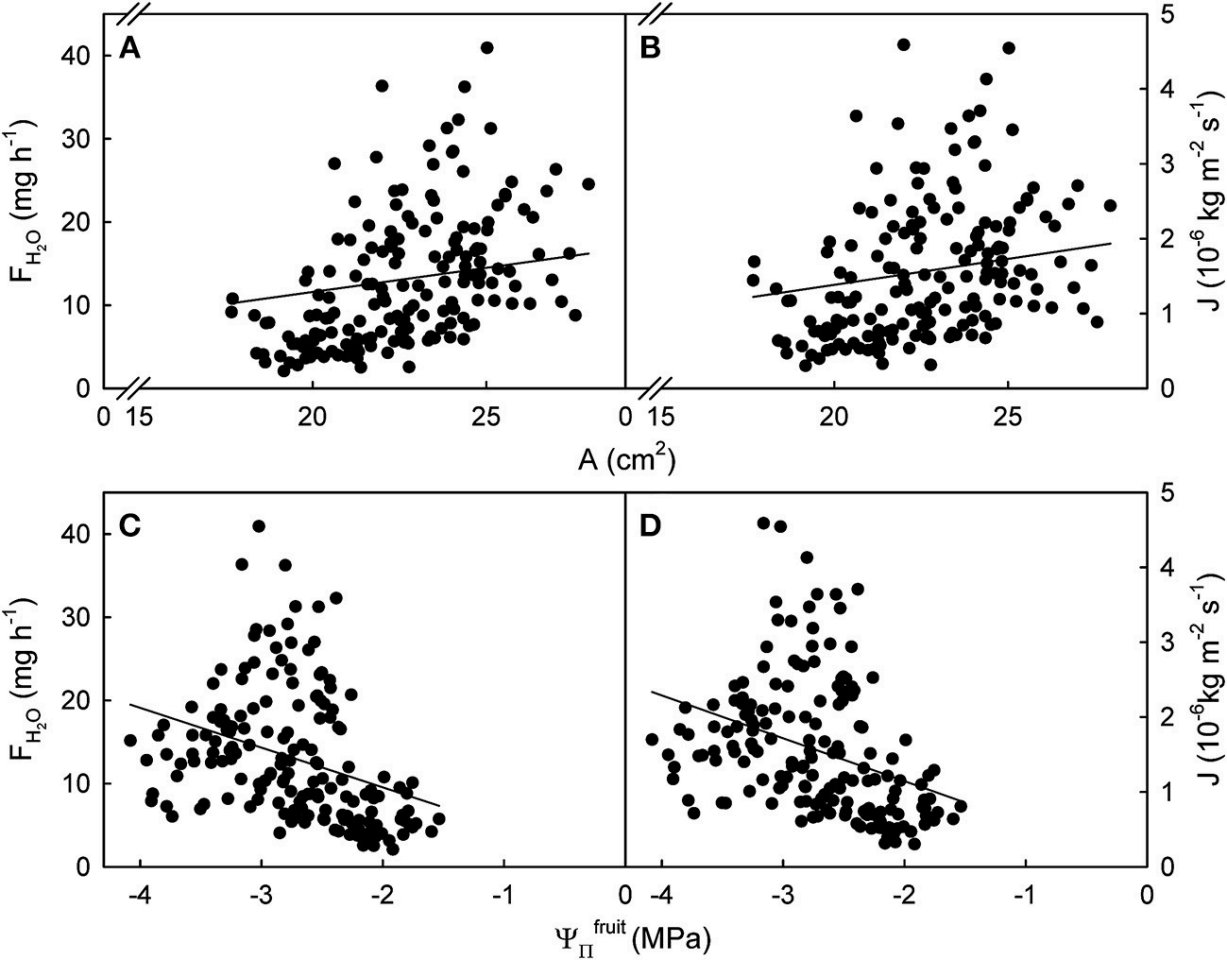
Relationship between the rate of water uptake [*F*_*H*_2_*O*_; **(A,C)**] or the flux in water uptake [*J*; **(B,D)**] and the surface area (*A*) or the osmotic potential (${\text{Ψ}}_{\Pi }^{fruit}$) of mature sweet cherry fruit. The equations of the linear regression lines were: *F*_*H*_2_*O*_ (g h^−1^) = 1.71 (±0.24) × *A* (cm^2^) – 25.86 (±5.47); *r*^2^ = 0.23 **(A)**; *J* (× 10^−6^ kg m^−2^ s^−1^) = 0.15 (±0.03) × *A* (cm^2^) – 1.75 (± 0.66); *r*^2^ = 0.13 **(B)**; *F*_*H*_2_*O*_ (g h^−1^) = −4.78 (± 0.20) × ${\text{Ψ}}_{\Pi }^{fruit}$ (MPa); *r*^2^ = 0.14 **(C)**; *J* (× 10^−6^ kg m^−2^ s^−1^) = −0.57 (± 0.02) × ${\text{Ψ}}_{\Pi }^{fruit}$ (MPa); *r*^2^ = 0.12 **(D)**. The intercept terms for **(C,D)** were not significant. Data symbols represent individual fruit.

Data are presented as means ± standard error of means. Where error bars are not visible in a graph, they are smaller than the data symbols. The exception is Figure 1, where data points for individual fruit are shown. Data were examined using analysis of variance or regression analysis. Pairwise comparisons of treatment means were carried out using Tukey's Studentized range test (*P* ≤ 0.05, package multcomp 1.3–1, procedure glht, R version 3.3.2; R Foundation for Statistical Computing, Vienna, Austria). Regression analysis was performed using R (version 3.3.2) and SigmaPlot (version 12.5; Systat Software, San Jose, CA). Significance of coefficients of determination (r^2^) at p ≤ 0.05, 0.01, and 0.001 is indicated by ^*^, ^**^, and ^***^, respectively.

## Results

The relationships between *F*_*H*_2_*O*_ or *J* and *A* or ${\text{Ψ}}_{\Pi }^{fruit}$ were significant but highly variable, as indicated by coefficients of determination ranging from r^2^ = 0.12^***^ to r^2^ = 0.23^***^. Variability was particularly high for larger *A* and larger driving forces as indexed by more negative ${\text{Ψ}}_{\Pi }^{fruit}$. There was little difference in variability between the relationships of *A* or ${\text{Ψ}}_{\Pi }^{fruit}$ with *F*_*H*_2_*O*_ and *J* (Figure 1).

**Figure 2:**
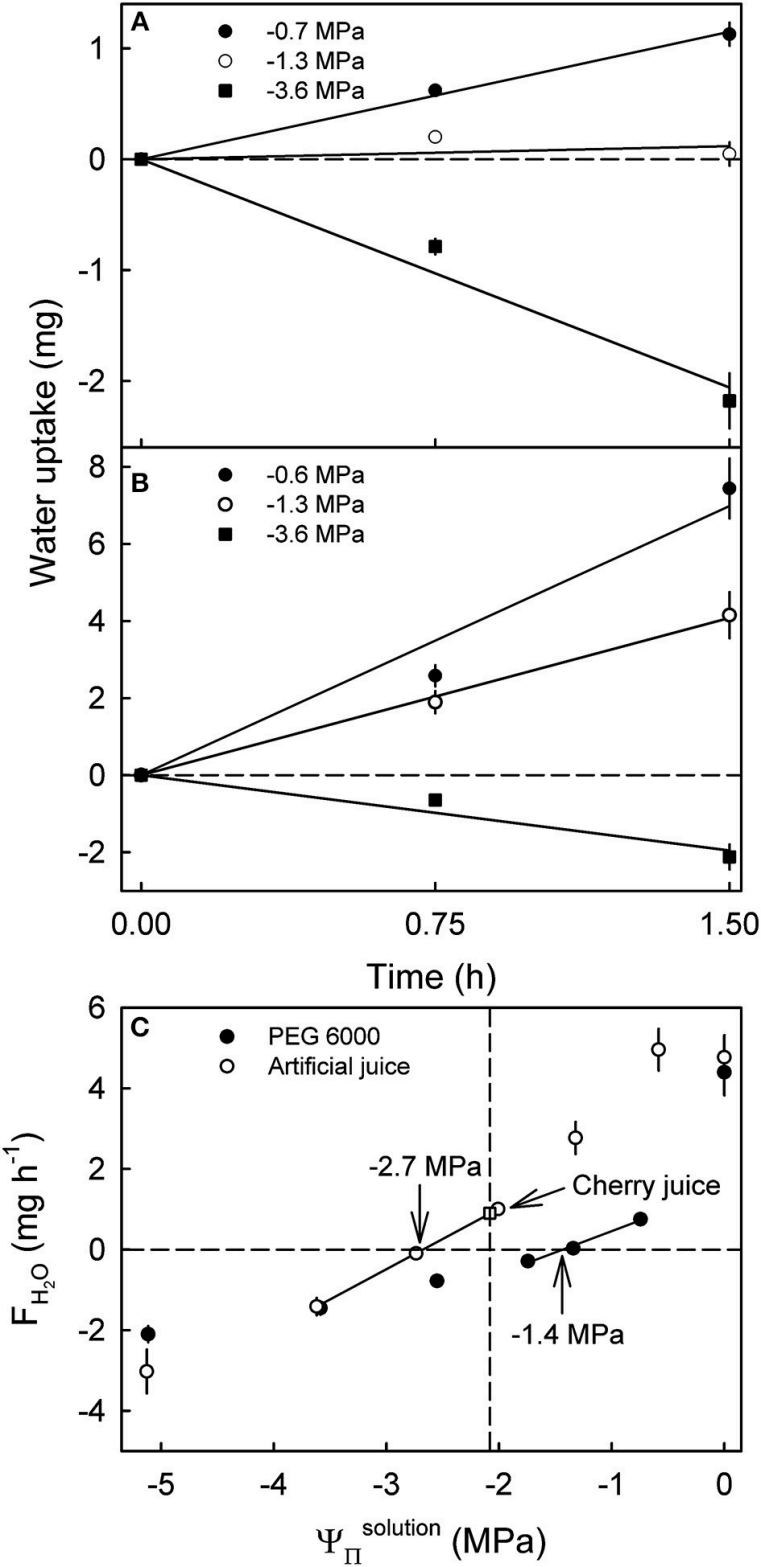
Effect of the osmotic potential (${\text{Ψ}}_{\Pi }^{solution}$) of polyethylene glycol 6000 (PEG 6000) **(A)** and artificial or natural sweet cherry juice **(B)** on the time course of water uptake and rates of water uptake (*F*_*H*_2_*O*_) **(C)**. Artificial cherry juice was prepared using the five most abundant osmolytes of sweet cherry fruit, i.e., glucose, fructose, sorbitol, malic acid, and potassium malate. The open square represents the water uptake from juice extracted from the same batch of fruit. The apparent fruit water potential (Ψ^*fruit*^) equals the ${\text{Ψ}}_{\Pi }^{solution}$ causing no change in fruit mass. The apparent Ψ^*fruit*^ is indicated by arrows. The dashed vertical line represents the ${\text{Ψ}}_{\Pi }^{fruit}$.

Varying the ${\text{Ψ}}_{\Pi }^{solution}$ of artificial juice or of PEG 6000 significantly affected water uptake. Uptake from hypotonic artificial sweet cherry juice and from hypotonic PEG 6000 solutions increased with time during a 1.5 h incubation at an approximately constant rate (Figures 2A,B). Making the ${\text{Ψ}}_{\Pi }^{solution}$ more negative, decreased rates of water uptake. At equal ${\text{Ψ}}_{\Pi }^{solution}$, the *F*_*H*_2_*O*_ from artificial juice exceeded that from isotonic PEG 6000 (Figure 2C). The only exceptions were hypertonic solutions, where *F*_*H*_2_*O*_ values from artificial juice were equal to those from PEG 6000 or higher. Interestingly, the apparent Ψ^*fruit*^ averaged −2.7 MPa for fruit incubated in artificial juice, but −1.4 MPa for that incubated in PEG 6000 (Figure 2C). At equal tonicity, there was no difference in *F*_*H*_2_*O*_ from artificial juice and “real” juice extracted from the same batch of fruit. When calculating the fruit Ψ_*P*_, an excessively high calculated fruit Ψ_*P*_ was obtained from Ψ^*fruit*^ determined in PEG 6000 (Ψ^*fruit*^ = −1.4 MPa, ${\text{Ψ}}_{\Pi }^{fruit}=$ −2.6 MPa, calculated fruit Ψ_*P*_ = 1.2 MPa). However, a negative fruit Ψ_*P*_ was calculated for Ψ^*fruit*^ determined in artificial juice (Ψ^*fruit*^ = −2.7 MPa, ${\text{Ψ}}_{\Pi }^{fruit}=$ −2.2 MPa, Ψ_*P*_ = −0.5 MPa). Most surprisingly, fruit incubated in its own juice or in artificial juice of the same tonicity as the fruits' juice had a positive *F*_*H*_2_*O*_ (Figure 2C).

The extremely high fruit Ψ_*P*_ calculated from Ψ^*fruit*^ determined in PEG 6000 was not unique to a specific cultivar but was also obtained in other sweet cherry cultivars (Table 1).

**Table 1:** Osmotic potential (${\text{Ψ}}_{\Pi }^{fruit}$), apparent fruit water potential (Ψ^*fruit*^) and the calculated fruit turgor (Ψ_*P*_) of selected cultivars of sweet cherry.

**Cultivar**	**${\text{Ψ}}_{\Pi }^{fruit}$ (MPa)**	**Apparent Ψ^*fruit*^ (MPa)**	**Calculated Ψ_*P*_ (MPa)**
Adriana	−2.1 ± 0.1	−1.4 ± 0.0	0.7 ± 0.1
Regina	−3.4 ± 0.1	−3.0 ± 0.2	0.4 ± 0.3
Sam	−3.0 ± 0.1	−2.6 ± 0.3	0.4 ± 0.3
Samba	−2.8 ± 0.1	−2.4 ± 0.0	0.4 ± 0.3
Grand mean	−2.8 ± 0.2	−2.4 ± 0.3	0.5 ± 0.1

*Fruit were incubated in PEG 6000 solutions ranging in osmotic potential from −5.4 to 0 MPa. The rate of water uptake was determined gravimetrically. The apparent Ψ^fruit^ was calculated as the point of zero mass change. The calculated Ψ_P_ was determined as the difference between ${\text{Ψ}}_{\Pi }^{fruit}$ and the apparent Ψ^fruit^*.

Water uptake from its own juice was also observed in other sweet cherry cultivars and also in two out of three sour cherry cultivars (Table 2).

**Table 2:** Osmotic potentials (${\text{Ψ}}_{\Pi }^{fruit}$) and rates of water uptake (*F*_*H*_2_*O*_) from deionized water and from juice extracted from selected cultivars of sweet (*Prunus avium*) and sour cherry (*Prunus cerasus*).

**Species**	**Cultivar**	**${\text{Ψ}}_{\Pi }^{fruit}$ (MPa)**	***F*_*H*_2_*O*_ (mg h^−1^)**	
			**Water**	**Juice**
*Prunus avium*	Adriana	−1.5	4.4 ± 0.4	1.0 ± 0.1
	Hedelfinger	−2.9	12.0 ± 1.1	3.2 ± 0.4
	Regina	−2.6	8.6 ± 1.1	1.3 ± 0.2
	Samba	−2.4	9.9 ± 1.1	1.5 ± 0.1
	Schneiders Späte Knorpel	−2.6	15.1 ± 2.5	1.4 ± 0.2
*Prunus cerasus*	Achat	−3.0	7.4 ± 0.8	−0.1 ± 0.0
	Morellenfeuer	−2.8	21.6 ± 1.8	2.8 ± 0.2
	Ungarische Traubige	−2.9	11.5 ± 1.1	0.3 ± 0.1
Grand mean		−2.5	11.3 ± 0.6	1.4 ± 0.1

*Cherry juice was extracted from the same batch of fruit*.

Heat treatment had no effect on the water uptake from juice indicating that artifacts that might result from enzymatic activity in the juice and that may have changed the ${\text{Ψ}}_{\Pi }^{solution}$ can be excluded as factor. Heating juice to 60°C produced the same *F*_*H*_2_*O*_ (1.3 ± 0.2 mg h^−1^) as that from non-heated juice (1.0 ± 0.1 mg h^−1^) or from artificial juice of the same tonicity (1.4 ± 0.1 mg h^−1^).

The pH of natural juice or that of artificial juice had no effect on *F*_*H*_2_*O*_. For natural juice of pH 4.2 and pH 7.0, the *F*_*H*_2_*O*_ were 1.1 ± 0.2 mg h^−1^ and 1.2 ± 0.2 mg h^−1^, respectively, and for artificial juice 1.1 ± 0.2 mg h^−1^ at pH 4.2 and 1.3 ± 0.2 mg h^−1^ at pH 7.0.

**Figure 3:**
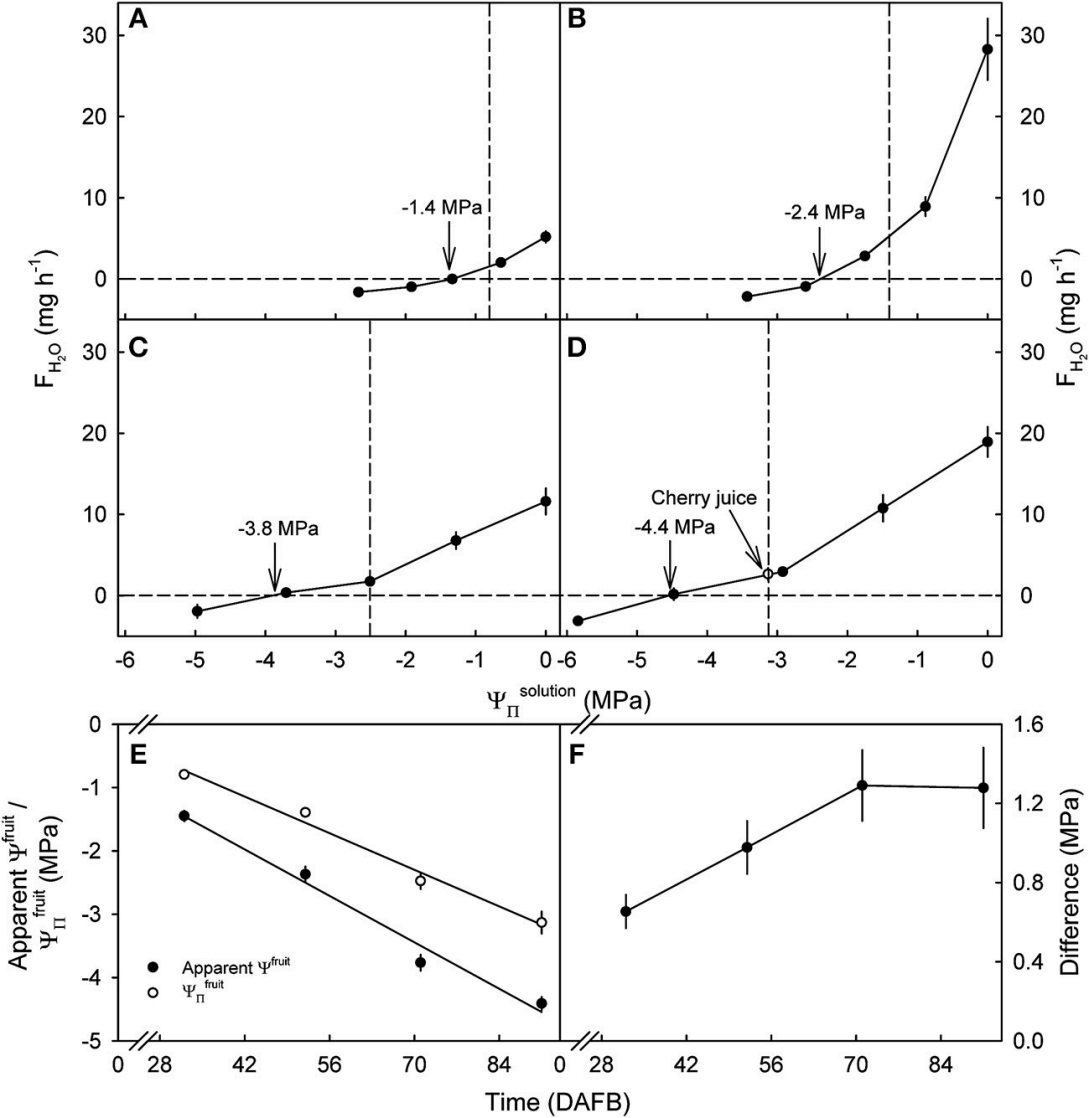
Effect of the osmotic potential of glucose incubation solutions (${\text{Ψ}}_{\Pi }^{solution}$) on the rates of water uptake (*F*_*H*_2_*O*_) and the apparent fruit water potential (Ψ^*fruit*^) of developing sweet cherry fruit [**(A)** 32, **(B)** 52, **(C)** 71, and **(D)** 91 days after full bloom (DAFB)]. The open circle [see arrow in **(D)**] represents water uptake from juice extracted from the same batch of fruit. **(E)** Apparent Ψ^*fruit*^ and osmotic potentials (${\text{Ψ}}_{\Pi }^{fruit}$) in the course of development. **(F)** Difference between apparent Ψ^*fruit*^ and ${\text{Ψ}}_{\Pi }^{fruit}$ in the course of development. The apparent Ψ^*fruit*^ equals the ${\text{Ψ}}_{\Pi }^{solution}$ causing no change in fruit mass and is indicated by vertical arrows **(A–D)**. Vertical dashed lines in **(A–D)** indicate the ${\text{Ψ}}_{\Pi }^{fruit}$.

Water uptake decreased, becoming a water loss at all developmental stages as the value of ${\text{Ψ}}_{\Pi }^{solution}$ of the glucose solutions became more negative (Figure 3). The apparent Ψ^*fruit*^ and the ${\text{Ψ}}_{\Pi }^{fruit}$ became more negative as development progressed (Figures 3A–E). Interestingly, the apparent Ψ^*fruit*^ was always more negative than the ${\text{Ψ}}_{\Pi }^{fruit}$ implying a negative calculated fruit Ψ_*P*_. The difference between the apparent Ψ^*fruit*^ and the ${\text{Ψ}}_{\Pi }^{fruit}$ increased up to 71 DAFB and, thereafter, remained constant at about −1.3 MPa (Figure 3F). At equal tonicity rates of uptake did not differ between fruit incubated in juice and in glucose solutions (Figure 3D).

There was no effect of the history of transpiration of the fruit on the difference between the ${\text{Ψ}}_{\Pi }^{fruit}$ determined in the water vapor osmometer and the apparent Ψ^*fruit*^ determined in the incubation assays. Holding fruit for 48 h at ~100% RH slightly increased the ${\text{Ψ}}_{\Pi }^{fruit}$ from −2.7 to −2.6 MPa and the Ψ^*fruit*^ from −3.8 to −3.7 MPa. Fruit held at ~0% RH lost 1.4 g water and decreased in ${\text{Ψ}}_{\Pi }^{fruit}$ from −2.7 to −3.3 MPa and in the apparent Ψ^*fruit*^ from −3.8 to −4.4 MPa. The difference between the ${\text{Ψ}}_{\Pi }^{fruit}$ and the apparent Ψ^*fruit*^ in both treatments and the control remained constant at 1.1 MPa (Table 3). Holding fruit under transpiring conditions (~0% RH) for up to 6.4 d produced a mass loss of up to 2.2 g and a proportional decrease in the value of ${\text{Ψ}}_{\Pi }^{fruit}$ as compared to fruit held at 100% RH (data not shown). However, despite the high mass loss at ~0% RH, there was no detectable horizontal gradient in ${\text{Ψ}}_{\Pi }^{fruit}$ within the equatorial plane between the fruit's outer and inner flesh. When holding fruit under non-transpiring conditions (~100% RH), mass and ${\text{Ψ}}_{\Pi }^{flesh}$ remained constant (data not shown).

**Table 3:** Osmotic potential (${\text{Ψ}}_{\Pi }^{fruit}$), apparent fruit water potential (Ψ^*fruit*^) and the calculated turgor (Ψ_*P*_) of sweet cherry fruit held for 4 d at ~100% RH or ~0% RH at 22°C.

**Treatment**	**RH (%)**	**${\text{Ψ}}_{\Pi }^{fruit}$ (MPa)**	**Apparent Ψ^*fruit*^ (MPa)**	**Calculated Ψ_*P*_ (MPa)**
0 d	–	−2.7 ± 0.1	−3.8 ± 0.1	−1.1 ± 0.1
4 d	100	−2.6 ± 0.1	−3.7 ± 0.3	−1.1 ± 0.3
4 d	0^*^	−3.3 ± 0.1	−4.4 ± 0.3	−1.1 ± 0.4

*Fruit were incubated in glucose solutions ranging in osmotic potential from −5.8 to 0 MPa. The rate of water uptake was determined gravimetrically. The apparent Ψ^fruit^ was calculated as the point of zero mass change. The Ψ_P_ was calculated as the difference between ${\text{Ψ}}_{\Pi }^{fruit}$ and the apparent Ψ^fruit^*.

* *Mass loss averaged 1.4 g per fruit*.

**Figure 4:**
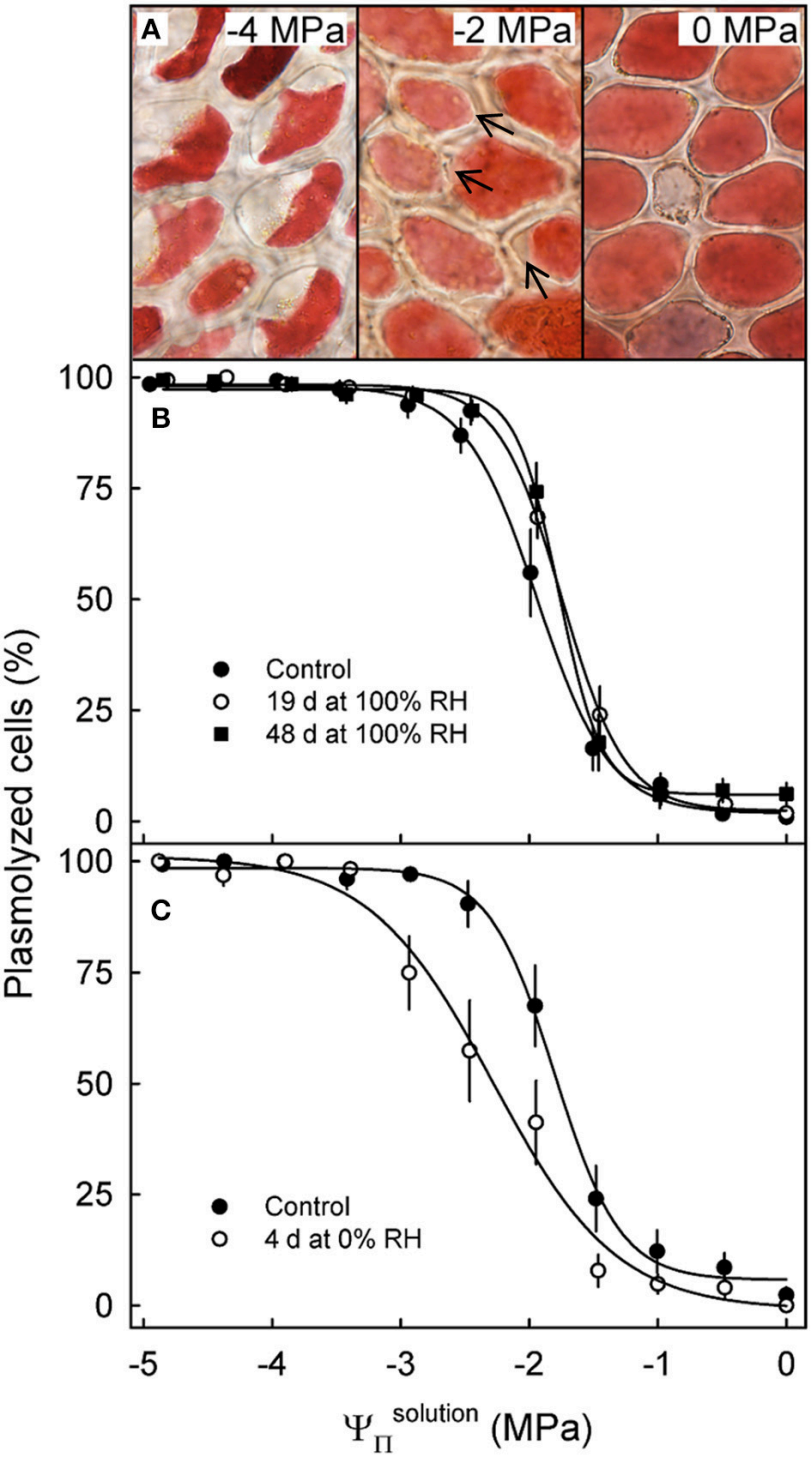
Effect of the osmotic potential of sucrose solutions (${\text{Ψ}}_{\Pi }^{solution}$) on plasmolysis of epidermal cells. Arrows indicate the onset of plasmolysis **(A)**. Effect of ${\text{Ψ}}_{\Pi }^{solution}\;$on the percentage of plasmolyzed epidermal cells of sweet cherry fruit held under non-transpiring conditions **(B)** or transpiring conditions **(C)**. Non-transpiring and transpiring conditions were imposed on the fruit to equilibrate (non-transpiring) or to induce a gradient (transpiring) in the osmotic potential between fruit and skin. Fruit was held under non-transpiring conditions (~100% RH) for up to 48 d or under transpiring conditions (~0% RH) for 4 d.

Incubating skin sections in sucrose solutions resulted in plasmolysis depending on the concentration used (Figure 4A). The percentage of plasmolyzed epidermal cells increased in a sigmoidal fashion as ${\text{Ψ}}_{\Pi }^{solution}$ decreased (Figures 4A,B). The osmotic potential at 50% plasmolysis represents the ${\text{Ψ}}_{\Pi }^{skin}$, which was always less negative than the ${\text{Ψ}}_{\Pi }^{flesh}$, as indexed by the ${\text{Ψ}}_{\Pi }^{fruit}$. Holding fruit for up to 48 d at ~100% RH slightly reduced, but did not eliminate the difference between ${\text{Ψ}}_{\Pi }^{flesh}$ and ${\text{Ψ}}_{\Pi }^{skin}$, the former being approximately equal to the ${\text{Ψ}}_{\Pi }^{fruit}\;$(Table 4). Fruit held at ~0% RH for 4 d lost 1.4 g and had slightly more negative ${\text{Ψ}}_{\Pi }^{flesh}$ and ${\text{Ψ}}_{\Pi }^{skin}$ (Figure 4C). The difference between the ${\text{Ψ}}_{\Pi }^{flesh}$ and ${\text{Ψ}}_{\Pi }^{skin}$, however, remained constant.

**Table 4:** Difference between the osmotic potential of the flesh (${\text{Ψ}}_{\Pi }^{flesh}$) and the skin (${\text{Ψ}}_{\Pi }^{skin}$) of mature sweet cherry fruit after holding up to 48 d at ~100% RH or for 4 d at ~0% RH at 22°C.

**Treatment**	**RH (%)**	**${\text{Ψ}}_{\Pi }^{fruit}$ (MPa)**	**${\text{Ψ}}_{\Pi }^{skin}$ (MPa)**	**${\text{Ψ}}_{\Pi }^{fruit}$**-**${\text{Ψ}}_{\Pi }^{skin}$ (MPa)**
0 d	–	−2.6 ± 0.1	−1.9 ± 0.1	−0.7 ± 0.1
19 d	100	−2.4 ± 0.1	−1.7 ± 0.0	−0.7 ± 0.1
48 d	100	−2.2 ± 0.1	−1.8 ± 0.0	−0.4 ± 0.1
0 d	–	−2.7 ± 0.1	−1.8 ± 0.1	−0.9 ± 0.1
4 d	0^*^	−3.0 ± 0.0	−2.3 ± 0.1	−0.8 ± 0.1

*The ${\text{Ψ}}_{\Pi }^{skin}$ is estimated from the osmotic potential of the incubation solution yielding 50% plasmolysis of the epidermal cells (see Figure 4)*.

* *Mass loss averaged 1.4 g per fruit*.

**Figure 5:**
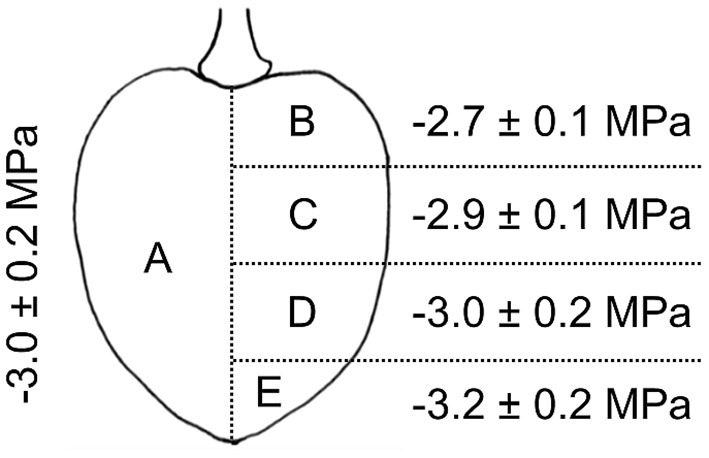
Osmotic potential of different parts of the sweet cherry fruit. Fruit was cut longitudinally along the pedicel stylar scar axis in half. Osmotic potentials were determined for juice extracted from one of the halves **(A)** and for four horizontal sections of the other half, i.e., sections comprising the regions of the pedicel end **(B)**, the pedicel center **(C)**, the stylar scar center **(D)**, or the stylar scar **(E)**.

The ${\text{Ψ}}_{\Pi }^{fruit}$ differed between different parts of the fruit. A significant vertical gradient in ${\text{Ψ}}_{\Pi }^{fruit}$ was present in the flesh from the pedicel region (−2.7 ± 0.1 MPa) to the stylar scar region (−3.2 ± 0.2 MPa). Thus, the pedicel region had a less negative and the stylar scar region a more negative ${\text{Ψ}}_{\Pi }^{fruit}$ value compared to the mean ${\text{Ψ}}_{\Pi }^{fruit}$ of −3.0 ± 0.2 MPa (Figure 5).

**Figure 6:**
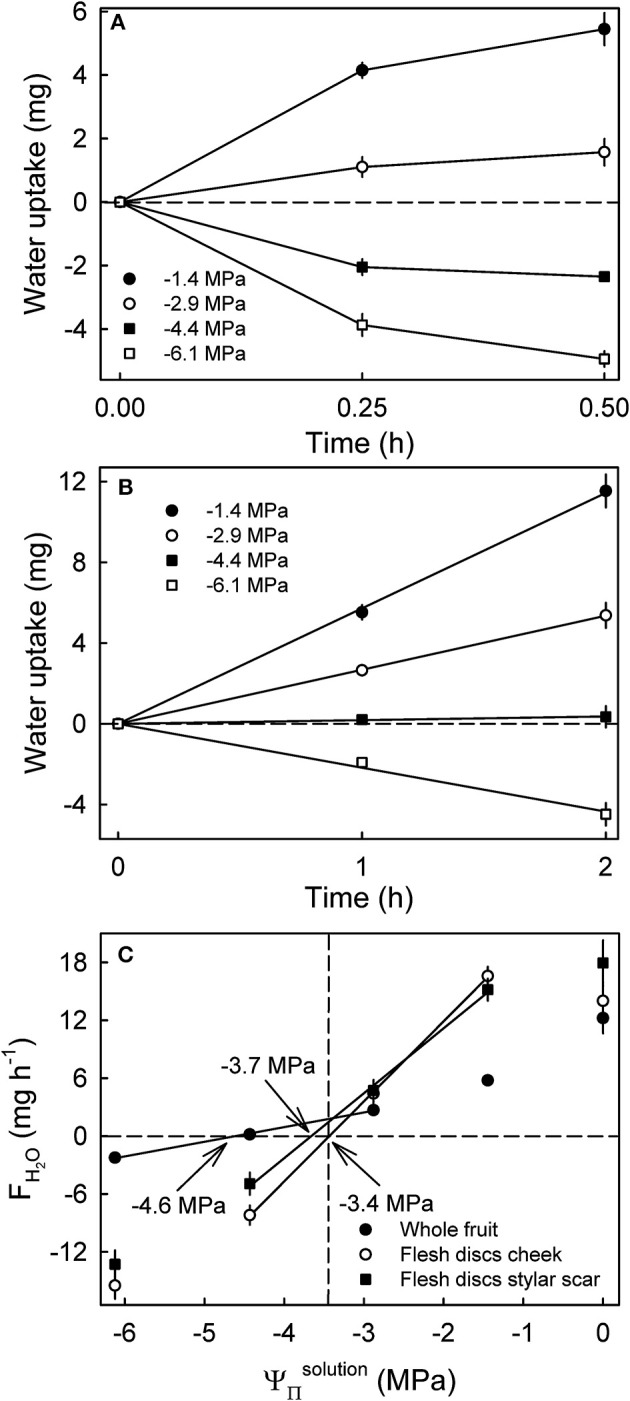
Time course of water uptake into discs excised from the flesh of mature sweet cherry fruit **(A)** and whole fruit **(B)**. **(C)** Rates of water uptake (*F*_*H*_2_*O*_) as affected by the osmotic potentials of the glucose incubation solutions (${\text{Ψ}}_{\Pi }^{solution}$). The apparent fruit water potential equals the water potential of a solution causing no change in fruit mass and is indicated by arrows. The dashed vertical line represents the osmotic potential of the fruits' juice.

Water uptake into flesh discs leveled off after 0.25 h indicating a decrease in *F*_*H*_2_*O*_ (Figure 6A), whereas cumulative uptake into whole fruit increased linearly (i.e., a constant rate of uptake) up to 2 h (Figure 6B). The apparent water potentials were −3.4 MPa for flesh discs excised from the cheek, −3.7 MPa for those from the stylar scar region and −4.6 MPa for whole fruit (Ψ^*fruit*^) from the same batch (Figure 6C). Thus, the apparent water potentials for the cheek matched the mean ${\text{Ψ}}_{\Pi }^{fruit}$ of the juice (−3.4 MPa), whereas the apparent water potential for the stylar scar was about 10% more negative and the apparent Ψ^*fruit*^ was far more negative than the mean ${\text{Ψ}}_{\Pi }^{fruit}$ of the juice (Figure 6C).

**Figure 7:**
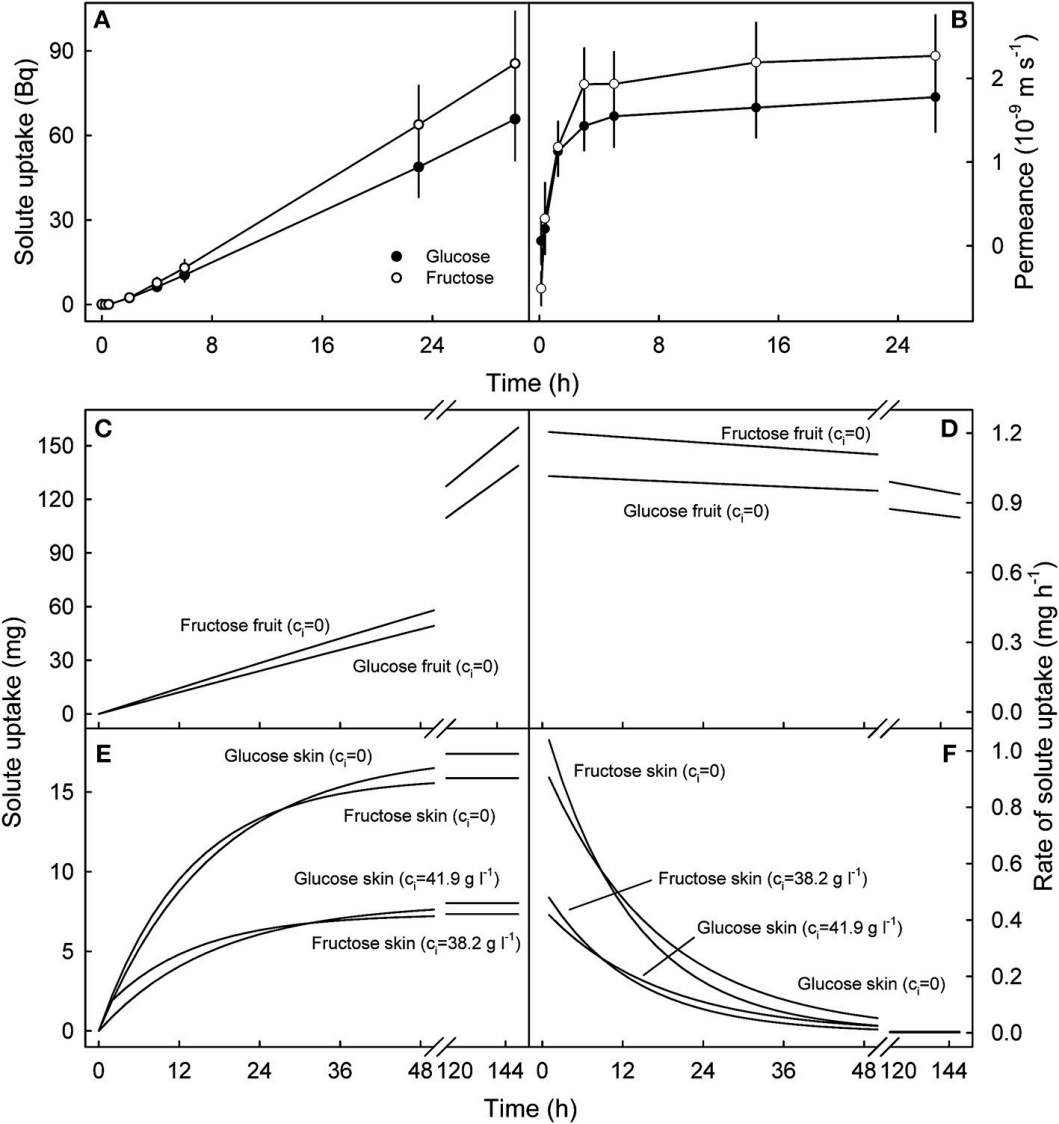
Time course of cumulative uptake of ^14^C-glucose and ^14^C-fructose through the sweet cherry fruit skin **(A)** and of the change in skin permeance (*P*) with time **(B)**. **(C–F)**. Simulated uptake of glucose and fructose into the fruit **(C,D)** and into the skin compartment **(E,F)**. **(C,D)** Cumulative uptake and rate of uptake into an intact sweet cherry that does not contain any glucose and fructose, i.e. the internal concentrations of glucose and fructose (*c*_*i*_) equal zero (*c*_*i*_ = 0). **(E,F)** Cumulative uptake and rate of uptake into the skin compartment of a sweet cherry fruit. The skin was assumed to contain no glucose or fructose (*c*_*i*_ = 0) or glucose and fructose at concentrations corresponding to those at the skin water potentials estimated by plasmolysis (*c*_*i*_ = 41.9 g l^−1^ for glucose, *c*_*i*_ = 38.2 g l^−1^ for fructose). The simulations were performed using the following parameters: *P*_*glucose*_: 1.6 × 10^−9^ m s^−1^, *P*_*fructose*_: 2.1 × 10^−9^ m s^−1^, fruit mass 10 g, fruit surface area 22.4 cm^2^, skin thickness 100 μm, density 1 kg l^−1^, no pit, concentration of incubation solution: *c*_*o*_ = 77.8 g l^−1^ for glucose, *c*_*o*_ = 70.9 g l^−1^ for fructose.

Uptake of ^14^C-glucose and ^14^C-fructose through the fruit skin reached a steady state after a lag phase of about 2 h, so the cumulative value increased linearly with time indicating the permeance approached a constant value at 1.6 ± 0.4 × 10^−9^ m s^−1^ for glucose and 2.2 ± 0.5 × 10^−9^ m s^−1^ for fructose (Figures 7A,B). Subsequent simulations demonstrated that glucose and fructose uptake into a 10 g sweet cherry fruit incubated in glucose and fructose concentrations that typically occur in the fruit's juice would also increase linearly over periods of about 150 h at approximately constant rates of between 0.9 and 1.2 mg h^−1^ provided that the fruit contained water only (Figures 7C,D). In contrast, simulations using the fruit skin as the receiver compartment, revealed that penetration leveled off within approximately 48 h for fruit skins containing no glucose or fructose and somewhat earlier for skins that contain both osmolytes at the concentrations assumed to be representative for the fruit skin (Figure 7E). For the latter two scenarios, rates of uptake were estimated at about 0.4 mg h^−1^ and 1 mg h^−1^ during the initial 1 h of the simulation, with little difference between the two carbohydrates (Figure 7F).

## Discussion

### A Sweet Cherry Is More Complex Than a Simple Osmometer

Our results demonstrate that a sweet cherry fruit behaves as an ideal osmometer only in some respects, but not in all. Consistent with being ideal osmometers is their response to incubation in solutions of various ${\text{Ψ}}_{\Pi }^{solution}$. For a given osmolyte or mixture of osmolytes, decreasing the ${\text{Ψ}}_{\Pi }^{solution}$ (i.e., making it more negative) decreases water uptake and *vice versa* for increases in ${\text{Ψ}}_{\Pi }^{solution}$. This response was obtained for all osmolytes we investigated, including for PEG 6000, artificial cherry juice and for glucose. It is also consistent with observations reported earlier for sweet cherries (Weichert and Knoche, 2006) and for grapes (Becker and Knoche, 2011) and currants (*Ribes*) (Khanal et al., 2011).

However, behaviors inconsistent with an ideal osmometer were:

1. The apparent Ψ^*fruit*^, determined as the point of zero net mass change for fruit incubated in various osmolytes, this point depended on the osmolyte used. This behavior was revealed both in this study and also in an earlier one (Weichert and Knoche, 2006). PEG 6000 yielded a less negative apparent Ψ^*fruit*^ than artificial juice. This response was not related to the low pH of the artificial juice but occurred also when the pH was adjusted to pH 7.0.
2. The difference between the value of apparent Ψ^*fruit*^ and ${\text{Ψ}}_{\Pi }^{fruit}$ (i.e., ΔΨ) has previously been considered to estimate fruit Ψ_*P*_. However, when subtracting the apparent Ψ^*fruit*^ (determined with PEG 6000 solutions) from ${\text{Ψ}}_{\Pi }^{fruit}$, excessively high values for the fruit Ψ_*P*_ are obtained. These are referred to here as the calculated fruit Ψ_*P*_. Literature data for sweet cherries and for other fruit crops yields values of the same order of magnitude (Measham et al., 2009; Table 5). However, experimentally determined values of fruit and cell Ψ_*P*_ using a pressure probe with mature fruit are negligibly low relative to values of ${\text{Ψ}}_{\Pi }^{solution}$ (<0.1 MPa for grapes, see Thomas et al., 2006, 2008; 0.008 to 0.064 MPa for sweet cherry, see Knoche et al., 2014; Schumann et al., 2014). For grape berries, the lack of a significant measured cell Ψ_*P*_, despite the very negative ${\text{Ψ}}_{\Pi }^{fruit}$, was accounted for by the presence of apoplastic solutes (Wada et al., 2008, 2009), possibly as a result of a compartmental breakdown (Lang and Düring, 1991). Whether the same explanation applies to sweet cherry is not known.
3. Quite unexpectedly, we recorded significant rates of water uptake by fruits incubated in juice extracted from others of the same batch. This counterintuitive observation is difficult to explain as it is almost axiomatic that a stable system (i.e., an isolated fruit held for some time under low-transpiration conditions) will be at Ψ^*fruit*^equilibrium throughout. This state of affairs is likely true, even though different compartments in the fruit could well-achieve this same uniform Ψ^*fruit*^ value via different balances of the water potential components. The full Ψ^*fruit*^ equation in this case is ${{\text{Ψ}}^{fruit}=\text{Ψ}}_{P}+{\text{Ψ}}_{\Pi }+{\text{Ψ}}_{M}$ where, as discussed, Ψ_*P*_ is negligible, i.e., some two orders of magnitude smaller than Ψ_Π_. Further, a Ψ_*M*_ does not occur in mature sweet cherry because gas/liquid interfaces are largely absent in mature sweet cherry. Certainly, in the complete absence of fruit Ψ_*P*_, the ${\text{Ψ}}^{fruit}={\text{Ψ}}_{\Pi }$ and one would expect no change at all in fruit mass with such an incubation. As it turned out, in this and our earlier study, the osmotic potential of the fruit tissues differed between skin and flesh (Grimm and Knoche, 2015) and also along the fruit axis (pedicel to stylar scar). This latter observation is new. Most surprisingly, these internal gradients are remarkably stable, largely independent of the transpiration history of the fruit and indicative of some sort of water compartmentation between tissues.

**Table 5:** Osmotic potential (${\text{Ψ}}_{\Pi }^{fruit}$), apparent fruit water potential (Ψ^*fruit*^) and the calculated fruit turgor (Ψ_*P*_) of different Ribes species and grape cultivars from the literature.

**Species**	**Cultivar**	**${\text{Ψ}}_{\Pi }^{fruit}$ (MPa)**	****Ψ^*fruit*^** (MPa)**	**Calculated Ψ_*P*_ (MPa)**	**References**
*Ribes nigrum*	Zema	−2.7 ± 0.0	−2.1 ± 0.2	0.5 ± 0.2	Khanal et al., 2011
*Rives uva-crispa*	Rote Triumph	−1.9 ± 0.0	−1.2 ± 0.0	0.7 ± 0.1	
*Ribes × nigridolaria*	Jostine	−2.5 ± 0.0	−1.9 ± 0.2	0.6 ± 0.2	
*Vitis vinifera*	Chardonnay	−3.9 ± 0.0	−1.7 ± 0.1	2.3 ± 0.1	Becker and Knoche, 2011
*Vitis vinifera*	Müller-Thurgau	−3.5 ± 0.0	−2.3 ± 0.1	1.2 ± 0.1	
*Vitis vinifera*	Riesling	−3.7 ± 0.0	−1.6 ± 0.0	2.1 ± 0.0	

*The rate of water uptake was determined gravimetrically by incubating fruit in PEG 6000 solutions with different osmotic potentials. The Ψ^fruit^ was calculated as the point of zero mass change. The calculated Ψ_P_ was obtained as the difference between ${\text{Ψ}}_{\Pi }^{fruit}$ and Ψ^fruit^*.

We conclude that the hypothesis that a sweet cherry behaves as an ideal osmometer must be rejected. Some other factors are clearly involved and a more complex model is required.

### The CM has a Reflection Coefficient < 1 for Small Osmolytes

An ideal osmometer allows penetration of the solvent, but totally excludes penetration by solutes (osmolytes). The results for cherry, however, demonstrate that the CM allows passage of some osmolytes from the incubation solution into the fruit. In membrane studies, the permeability to solutes relative to the permeability to solvents is characterized by the dimensionless reflection coefficient σ (Nobel, 1999). Thus, the value σ = 1 implies a membrane permeable only to water. While a σ < 1 implies some diffusional inflow and outflow of osmolytes depending on the magnitude and direction of their individual chemical potential gradients. This being in addition to diffusional inflow and outflow of water depending on the magnitude and direction of the water potential gradient (also a chemical potential gradient). Because, for a given membrane, the value taken by σ depends on the size of the osmolyte, the value of σ differs from solute to solute. For any osmolyte just too large to penetrate, the sweet cherry CM will have σ value of 1, and for those smaller than this σ < 1.

The penetration of glucose (180 g mol^−1^) and fructose (180 g mol^−1^) through the skin of the sweet cherry fruit indicates the size of these solutes is below the exclusion limit of polar pathways in the CM and hence, the value of σ for these osmolytes is σ < 1. Polar pathways allow penetration of small polar molecules through a lipoidal cuticle (Schönherr, 2006). They are not physical holes in the lipophilic polymer, but result from the orientation of polar functional groups in a hydrated cuticle. Our findings are consistent with the size exclusion limits of the sweet cherry CM estimated earlier (Weichert and Knoche, 2006). The largest penetrating osmolyte was sucrose that had a σ = 0.74 (MW 342 g mol^−1^), the smallest non-penetrating osmolyte was PEG 1500 (MW 1500 g mol^−1^) (Weichert and Knoche, 2006). Consequently, the PEG 6000 (mean MW 6000 g mol^−1^) also used in the present study is size-excluded (σ = 1).

### Uptake Into Fruit When Incubated in its Own Juice

The observation of fruit taking up water when incubated in its own juice is difficult to explain. This behavior was consistent in seven out of the eight cultivars examined. Artifacts due to the low pH of the juice or due to enzymatic processes in the expressed juice were excluded, since neither adjusting the pH to neutral nor inactivating enzymes by heating, eliminated water uptake from the presumably isotonic solution. Furthermore, there was no difference in uptake between natural juice, an isotonic artificial juice or an isotonic glucose solution. Hence, the unexpected behavior was reproducible and consistent. This behavior forces us to question the underlying assumption that the fruit is an osmotic cell (an ideal osmometer) with a uniform internal water potential.

The results from this and our previous study show that the value of ${\text{Ψ}}_{\Pi }^{skin}$ (−1.4 MPa) is less negative than that of ${\text{Ψ}}_{\Pi }^{flesh}$ (−2.6 MPa; Grimm and Knoche, 2015). In the absence of significant Ψ_*P*_ (0.008 to 0.064 MPa; Knoche et al., 2014; Schumann et al., 2014), spatial differences in ${\text{Ψ}}_{\Pi }^{fruit}$ thus represent differences in Ψ^*fruit*^. Furthermore, these spatial differences increase during fruit development and are largely independent of the transpiration history of the fruit. Across five cultivars, the difference in osmotic potentials between ${\text{Ψ}}_{\Pi }^{flesh}$ and ${\text{Ψ}}_{\Pi }^{skin}$ averaged 1.1 MPa (Grimm and Knoche, 2015). Thus, incubating a fruit in its own juice (*in vitro*), subjected the skin to the same water potential gradient that prevailed between ${\text{Ψ}}_{\Pi }^{skin}$ and ${\text{Ψ}}_{\Pi }^{flesh}$ (*in vivo*). Furthermore, a concentration difference and, hence, a chemical potential difference existed between the osmolytes in the skin and those in the donor solution. The consequences of these osmolyte gradients are 2-fold:

(1) We know that osmolytes having σ < 1 for sweet cherry skin will diffuse from the donor solution into the fruit. Glucose and fructose, which together account for 78.7% of the osmolytes in sweet cherry juice (Herrmann, 2001), both exhibit σ < 1 for sweet cherry skin. Using the permeances determined herein, the rates of penetration of glucose and fructose into the skin compartment during the first hour of an uptake experiment was estimated between 0.4 and 1 mg h^−1^ each for glucose and fructose, respectively, depending on the concentrations of glucose and fructose in the fruit skin. These rates are of the same magnitude as those measured as being taken from the juice, e.g., about 1.4 ± 0.1 mg h^−1^. Also consistent with this explanation is the observation that the value of ${\text{Ψ}}_{\Pi }^{solution}$ approximated to ${\text{Ψ}}_{\Pi }^{fruit}$ when the skin compartment was “eliminated” by incubating flesh discs excised from the cheek in a glucose solution. The second consequence would be an efflux of osmolytes from the skin into the donor solution if the latter contained water only or PEG 6000 which is discussed in detail below.

In addition, the axial gradient in tissue water potential along the pedicel/stylar scar axis could also contribute to water uptake from its own juice provided there was a bias between the permeance of the fruit skin for water uptake and the water potential gradient driving uptake. This is indeed the case because the stylar scar region has a more negative osmotic potential than the average fruit juice and a higher skin permeance than the average fruit surface due to the high frequency of microcracks in this region (Peschel and Knoche, 2005).

### Unrealistic **Ψ*^fruit^*** Using PEG 6000 as Incubation Solution

Because sweet cherry fruit skin has a σ = 1 for PEG 6000 (Weichert and Knoche, 2006), PEG 6000 should be an ideal osmolyte. However, our data demonstrate that values of Ψ^*fruit*^ obtained using PEG 6000 are unrealistic and yield excessively high calculated Ψ_*P*_ in this and earlier studies. Several factors may be involved.

When a fruit is incubated in a solution of PEG 6000, while water potential equilibrium may prevail between inside and outside (if the PEG 6000 concentration is chosen appropriately), there will be sharp concentration differences across the skin between the osmotic moieties in the fruit and the outside—initially there will be zero concentrations of these moieties outside. Hence, there will be an outward diffusion of the inside osmolytes for all those for which the skin offers σ < 1 (i.e., glucose and fructose, for which the outward diffusions will be −0.5 and −0.6 mg h^−1^, respectively). Meanwhile, there will be no inward diffusion of PEG 6000 (because, for it σ = 1). As a consequence, there will be an outward diffusion of water so Ψ^*fruit*^ will seem to be at a less negative osmotic potential than it really is. This diffusional loss of these moieties will also occur when a fruit is incubated in water. Preliminary experiments indeed revealed a loss of osmolytes from mature sweet cherry fruit to a water incubation solution that amounted on average to 0.16 mg h^−1^ (range 0.05–0.36). At present the osmolytes diffusing from the fruit into the incubation solution have not been identified, but the retention times in HPLC analyses of some of the unknown moieties corresponded to those of glucose and fructose—two major osmolytes of sweet cherry. A leakage of osmolytes having a σ < 1 would displace the water uptake curves and as a consequence, contribute to an overestimation of the Ψ^*fruit*^.

It may be argued that the excessively high (less negative) Ψ^*fruit*^ obtained in PEG 6000 are an artifact due to the high viscosity of the PEG 6000 solutions. Viscosities in the range of the PEG 6000 solutions used in these experiments reduce the rates of water uptake by 40–80% (Weichert and Knoche, 2006; Regupathi et al., 2009). A concentration dependent viscosity would explain the lack of a linear relationship between the ${\text{Ψ}}_{\Pi }^{solution}$ of PEG 6000 and the resulting rates of water uptake. However, viscosity would not affect the ${\text{Ψ}}_{\Pi }^{solution}$ at zero uptake.

Stomatal penetration can also be excluded as a factor. First, the critical surface tension (γ_crit_) of the fruit surface was reported at 24.9 mN m^−1^ (Peschel et al., 2003). Solutions having a surface tension below γ_crit_ will overcome capillary exclusion and may penetrate open stomata (Schönherr and Bukovac, 1972) and PEG 6000 (all other solutes) are likely to have a higher surface tension. Second, even if stomatal penetration occurred, this would only affect the rate of uptake, but not the water potential at which zero uptake occurs. Hence, stomatal penetration cannot account for the observed phenomenon.

## Conclusions

A sweet cherry is more complex than a simple osmometer. The CM has a σ < 1 for a number of common solutes including glucose and fructose. In addition, the osmolytes are not uniformly distributed within the fruit, resulting in water potential gradients from the less negative pedicel region to the more negative stylar scar region and from the less negative skin to the more negative flesh. As a consequence, the value of Ψ^*fruit*^ determined by incubating a fruit in a solution of osmolytes is potentially inaccurate as it is an average value. Also because of solute diffusion through the skin it will lead to an underestimation of the value of Ψ^*fruit*^ for small osmolytes (i.e., glucose) and an overestimation of the Ψ^*fruit*^ for large osmolytes (i.e., PEG 6000).

## Data Availability

The raw data supporting the conclusions of this manuscript will be made available by the authors, without undue reservation, to any qualified researcher.

## Author Contributions

MK obtained the funds to support the study. AW, EG, and MK planned the experiments. AW and EG conducted the experiments. AW, EG, and MK analyzed the data and AW and MK wrote, revised and edited the manuscript.

### Conflict of Interest Statement

The authors declare that the research was conducted in the absence of any commercial or financial relationships that could be construed as a potential conflict of interest.

## Abbreviations

*A*: surface area
CM: cuticular membrane
DAFB: days after full bloom
ES: epidermal skin segment
*F*_*H*_2_*O*_: rate of water uptake
*F*_*CHO*_: rate of carbohydrate uptake
*J*: flux density
*P*_*f*_: osmotic water permeability
*P*: Permeance
PEG: polyethylene glycol
γ_crit_: critical surface tension
ΔΨ: water potential difference
Ψ_*P*_: turgor
${\text{Ψ}}_{\Pi }^{flesh}$: osmotic potential of the flesh
Ψ^*fruit*^: fruit water potential
${\text{Ψ}}_{\Pi }^{fruit}$: osmotic potential of the fruit
${\text{Ψ}}_{\Pi }^{skin}$: osmotic potential of the skin
${\text{Ψ}}_{\Pi }^{solution}$: osmotic potential of the incubation solution.
